# Methylglyoxal-Dependent Glycative Stress Is Prevented by the Natural Antioxidant Oleuropein in Human Dental Pulp Stem Cells through Nrf2/Glo1 Pathway

**DOI:** 10.3390/antiox10050716

**Published:** 2021-05-01

**Authors:** Simona Delle Monache, Fanny Pulcini, Roberta Frosini, Vincenzo Mattei, Vincenzo Nicola Talesa, Cinzia Antognelli

**Affiliations:** 1 Department of Biotechnological and Applied Clinical Sciences, University of L’Aquila, 67100 L’Aquila, Italy; simona.dellemonache@univaq.it (S.D.M.); fanny.pulcini@graduate.univaq.it (F.P.); 2 Department of Medicine and Surgery, University of Perugia, 06123 Perugia, Italy; roberta.frosini@unipg.it (R.F.); vincenzo.talesa@unipg.it (V.N.T.); 3 Biomedicine and Advanced Technologies Rieti Center, “Sabina Universitas”, 02100 Rieti, Italy; v.mattei@sabinauniversitas.it; 4 Department of Experimental Medicine, “Sapienza” University of Rome, 00161 Rome, Italy

**Keywords:** methylglyoxal, oleuropein, dental pulp stem cells, glyoxalase 1, Nrf2, glycative stress, inflammation, oxidative stress, apoptosis

## Abstract

Methylglyoxal (MG) is a potent precursor of glycative stress (abnormal accumulation of advanced glycation end products, AGEs), a relevant condition underpinning the etiology of several diseases, including those of the oral cave. At present, synthetic agents able to trap MG are known; however, they have never been approved for clinical use because of their severe side effects. Hence, the search of bioactive natural scavengers remains a sector of strong research interest. Here, we investigated whether and how oleuropein (OP), the major bioactive component of olive leaf, was able to prevent MG-dependent glycative stress in human dental pulp stem cells (DPSCs). The cells were exposed to OP at 50 µM for 24 h prior to the administration of MG at 300 µM for additional 24 h. We found that OP prevented MG-induced glycative stress and DPSCs impairment by restoring the activity of Glyoxalase 1 (Glo1), the major detoxifying enzyme of MG, in a mechanism involving the redox-sensitive transcription factor Nrf2. Our results suggest that OP holds great promise for the development of preventive strategies for MG-derived AGEs-associated oral diseases and open new paths in research concerning additional studies on the protective potential of this secoiridoid.

## 1. Introduction

MG is a highly reactive dicarbonyl compound acting as a potent glycating agent. It can rapidly react with proteins, lipids, and nucleic acids, producing AGEs. A major and functionally important MG-derived AGE in physiological systems is 5-hydro-5-methylimidazolone (MG-H1). The primary defense against MG is represented by the GSH-dependent enzyme Glyoxalase 1 (Glo1). MG accumulation, due to its increased production or decreased detoxification by Glo1 or both, generates “dicarbonyl stress”. This condition frequently leads to increased intracellular levels of MG-derived AGEs, a state known as “glycative stress” [1]. In turn, glycative stress can lead to increased formation of reactive oxygen species (ROS) [2], activation of inflammatory pathways through the receptor for AGEs (RAGE) [3], activation of the mitochondrial pathway of apoptosis [4,5], and induction of epithelial-to mesenchymal transition [6]. Given that all these biological responses underpin the genesis of several human diseases, MG and MG-derived MG-H1, as well as Glo1, have been proved to play a crucial role in the etiogenesis of many diseases, including cancer [7,8,9], infertility [5,10], osteoporosis [2,11], obesity and diabetes [12]. At the same time, since MG is endogenously produced by cell metabolic pathways, mainly the glycolytic one [1], many metabolic disorders, primarily hyperglycemia and diabetes, among others, favor its production [13], which is in many cases co/responsible for several associated complications [14,15]. In addition to being produced endogenously, there are exogenous sources of MG, such as some foods, foodstuff autoxidation, food cooking and cigarette smoking [16,17]. Taking all this into account, preventing MG accumulation in cells is pharmacologically relevant in both prevention and treatment of diseases and/or their associated complications.

Mounting in vitro evidence, using mainly cell models from gingival connective tissue, suggests that MG and MG-derived AGEs are also involved in the pathogenesis of some oral diseases, such as gingivitis and periodontitis [18,19]. Moreover, some in vivo observations have pointed out the accumulation of MG in some biological fluids from the gingival crevicular fluid of chronic periodontitis patients [20] that can also be conveyed by bacterial infections [21], dietary compounds [22], and cigarette smoke or aerosols from nicotine delivery systems (NDS) (Electronic Cigarette and Heat-not-burn tobacco product IQOS) [23,24]. Notably, numerous clinical and experimental studies have highlighted the presence of a strong association between periodontitis, which represents the most common pathology of the oral cave in the adult population, and some systemic diseases, in particular, diabetes and obesity [25,26], which, as mentioned before, are characterized by increased levels of MG and MG-derived “glycative stress” onset.

Mesenchymal stem cells (MSCs) are multipotent stem cells. In adults, they retain the ability to differentiate into cells of several mesodermal tissues, including cartilage, bone, skeletal and cardiac muscles. A group of MSCs, with characteristics similar to bone marrow stem cells, has been recently isolated in the oral cavity, from the dental pulp, DPSCs [27]. DPSCs have a high capacity for self-renewal, large differentiative potential, and immuno-modulatory functions [28]. They are physiologically involved in the homeostasis of dentine and can differentiate into cementoblast-like cells, collagen-forming cells, with the ability to generate cement-like material from periodontal tissue, and odontoblasts. DPSCs are also very important for maintaining the vascular and nervous homeostasis of the teeth in addition to contributing to bone remodeling and tissue regeneration and repair [29]. Given their extreme ease of recovery and their high proliferative potential, DPSCs have greatly expanded the regenerative medicine horizons [27,30]. In consideration of the important functions of DPSCs in the oral cave health, factors that potentially may impair their viability and/or functionality, such as endogenous and/or exogenous MG, can profoundly influence the health state of the oral cave. Since, to date, there have been no studies regarding the potential detrimental effects of MG-derived dicarbonyl/glycative stress on DPSCs, we first wanted to investigate whether and through which mechanism this metabolite could affect DPSCs viability and functionality. Equally important in this context is the possibility of preventing the establishing of harmful MG-dependent dicarbonyl/glycative stress. Although synthetic agents able to rapidly trap MG into non-toxic compounds (and thereby avoiding MG-dependent glycative stress to be established) are known, they have never been approved for clinical use because of their side effects in human studies. Hence, the search of bioactive natural scavengers remains a sector of strong research interest.

OP is a phenolic compound found in olive fruit and the leaves of *Olea europaea* L., with a variety of pharmacological properties, above all antioxidant and anti-inflammatory ones [31]. To the best of our knowledge there is only one report describing the protective role of OP against MG-driven glycative stress in the hepatic cell line, HepG2 [32].

In the present study, we also wanted to investigate whether OP could act as an effective anti-glycative agent by protecting DPSCs from MG detrimental effects.

We found that MG induced in DPSCs glycative stress through the accumulation of its major AGE, MG-H1, that through desensitization of Nrf2/Glo1 pathway impaired their cell growth and functionality. More importantly, OP could protect DPSCs from MG-derived detrimental effects by restoring Nrf2/Glo1 pathway.

## 2. Materials and Methods

### 2.1. Materials

Reagents included: MG from Merck Spa (Milan, Italy), OP from Vinci-Biochem Srl (Florence, Italy). Nrf2 activator (Nrf2-A) was from EMD Millipore Corporation (Billerica, MA, USA). OP and Nrf2-A were dissolved in dimethyl sulfoxide (DMSO, Merck Spa, Milan, Italy) (final DMSO concentration in incubations = 0.01%). Controls contained an identical volume of DMSO vehicle. The bicinchoninic acid (BCA) kit for protein quantification was from Thermo Fisher Scientific (Monza, Italy). All the other reagents, where not otherwise specified, were acquired from Sigma-Aldrich (Milan, Italy).

### 2.2. Cell Culture and Treatments

Human dental pulp-derived stem cells (DPSCs) and human umbilical vein endothelial cells (HUVECs) were from Lonza (Walkersville, USA). DPSCs were cultured in Dental Pulp Stem Cell Medium Bullet kit (basal media and the necessary supplements) while HUVECs were grown in endothelial cell growth medium EGM-2 (basal medium, EBM-2, with the supplied frozen additives). Cells were maintained in a 37 °C incubator in a humidified atmosphere containing 5% CO_2_ according to the conditions indicated by the cell depository.

DPSC cells (fourth passage) were seeded at 8 × 10^4^ cells/well into 6-well plates and grown for 24 h to sub-confluence. In a first set of experiments, DPSCs were exposed to MG at 50, 150, 300 and 500 µM for 24 h. In a second set of experiments DPSCs were exposed to OP at 50 or 150 µM for 24 h prior to the administration of MG at 150 and 300 µM for additional 24 h. In a third set of experiments DPSCs were co-treated with 10 µM Nrf2-A and 50 µM OP for 16 h. After that, the Nrf-2 activator was removed, and OP was added back to the wells. Following a total 24 h of OP treatment, 300 µM MG was also added to the wells and left for additional 24 h.

### 2.3. Detection of MG-H1 Protein Adducts

MG-H1 protein adducts were measured by using a competitive enzyme-linked immunosorbent assay (ELISA) kit (DBA Italia Srl) according to the manufacturer’s instructions [9].

### 2.4. Protein Extraction

Total proteins extraction was performed by lysing the cells with radioimmunoprecipitation assay (RIPA) lysis buffer [4]. For nuclear extracts a FractionPREP Cell Fractionation kit (Biovision, VinciBiochem, Florence, Italy) was used [2].

### 2.5. Glyoxalase 1 (Glo1) Enzyme Activity and Protein Assay

Glo1 activity was assayed as previously described [9]. Protein concentration was determined with the BCA kit, using bovine serum albumin as a standard.

### 2.6. H_2_O_2_ and MDA Detection

H_2_O_2_ was measured as previously described [2]. The cellular concentration of MDA was determined as previously described [33].

### 2.7. GSH Detection

GSH was measured by using the GSH assay kit (colorimetric) (DBA Italia Srl) as per the manufacturer’s instructions.

### 2.8. Cell Proliferation Assay

Cell proliferation was evaluated by fixing cells with formalin 4% and staining with crystal violet (1%). Stained cells were solubilized using a solubilization solution containing 1% SDS and 50% methanol and the reading was carried out on 96-well multi-well (EuroClone, Milan, Italy) plates in a microplate reader at 595 nm.

### 2.9. Apoptosis Detection

Apoptosis was detected by caspase-3 activation using the specific human Caspase-3 (active) ELISA kit (Invitrogen, Milan, Italy) [2].

### 2.10. IL-1β, IL-6 and IL-8 Detection

IL-1β, IL-6 and IL-8 were measured by using specific, commercially available ELISA kits from ThermoFisher Scientific (IL-1 β) or U-CyTech, Biosciences for IL-6 and IL-8, according to the manufacturer’s instructions.

### 2.11. Nrf2, HO-1, Hsp70, Trx and γ-GCS Detection

Nrf2, HO-1, Hsp70, Trx and γ-GCS were measured by using specific, commercially available ELISA kits from Abcam (Prodotti Gianni, Milan) (Nrf2, HO-1, Hsp70 and γ-GCS) or Biomatik (Aurogene Srl, Rome) (Trx), according to the manufacturer’s instructions.

### 2.12. RNA Isolation, Reverse Transcription, and Real-Time Reverse Transcriptase-Polymerase Chain Reaction (RT-PCR) Analyses

Total cellular RNA was isolated using TRIzol Reagent (ThermoFisher Scientific, Milan). cDNA was then synthesized from 1 µg of RNA with the RevertAid™ H Minus First Strand cDNA Synthesis Kit (ThermoFisher Scientific, Milan). Gene expression versus β-actin was evaluated by RT-PCR on a MX3000P Real-Time PCR System (Agilent Technology, Milan, Italy). The sequences of the oligonucleotide primers are: Glo1 forward 5′-CTCTCCAGAAAAGCTACACTTTGAG-3′ and Glo1 reverse 5′-CGAGGGTCTGAATTGCCATTG-3′; RAGE forward 5′-CAGGATGAGGGGATTTTCCG-3′ and RAGE reverse 5′-AGGGACTTCACAGGTCAGGGTTAC-3′; β-actin forward: 5′-CACTCTTCCAGCCTTCCTTCC-3′ and β-actin reverse: 5′-ACAGCACTGTGTTGGCGTAC-3′. PCR primers were designed using Beacon Designer 4 software (version 4.0, Agilent Technology) from published sequence data stored in the NCBI database. PCR reactions were performed in a total volume of 20 µL, which contained 25 ng of cDNA, 1X Brilliant II SYBR^®^ Green QPCR Master Mix (Agilent Technology, Milan, Italy), ROX Reference Dye (Agilent Technology, Milan, Italy), and 600 nM of specific primers. The thermal cycling conditions were 1 cycle at 95 °C for 5 min followed by 45 cycles at 95 °C for 20 s and 60 °C for 30 s. To verify the possible co-amplification of unspecific targets, melting curves were performed for all of the primer pairs in standard conditions. The data required for carrying out a comparative analysis of gene expression were obtained by means of the 2^−(∆∆CT)^ method.

### 2.13. Preparation of DPSCs Conditioned Medium

DPSCs were seeded at 1 × 10^5^ cells/well into six-well plates. After attachment, cells were treated, as previously described, OP and MG alone or in combination and in the presence or not of Nrf-2 activator. To collect conditioned media, the medium was replaced, and cells were maintained for 24 h of incubation in serum free-medium (37 °C, in a humidified atmosphere containing 5% CO_2_). Supernatants were recovered, centrifuged at 2600× *g* at 4 °C for 10 min and the supernatant kept at −80 °C until needed for the experiments.

### 2.14. In Vitro Tubule Like-Formation and Stabilization Assay

The tube formation assay was carried out as previously described [34]. The degree of the angiogenic response was assessed after 16 h from the beginning of the treatment using an inverted phase contrast microscope by evaluating the number of branching points. Each well was photographed, and the respective acquired images quantified using an Image J analysis system. Branching index, obtained by measuring the number of junctions formed per vessel area, was used to quantify the levels of tube formation. To verify the ability of DPSCs to support HUVEC tube-like structures, we conducted a modified in vitro tube formation assay as previously described [27]. Briefly, HUVEC (1 × 10^4^ cells/well) were seeded in co-culture with DPSCs treated with different experimental conditions (MG, OP, Nrf2-A) and untreated on polymerized ECMatrix (Millipore) 15-well micro slides (10 μL/well) of IBIDI (Munich, Germany) following the manufacturer’s instructions in EGM-2 media (Promocell). Co-cultures of DPSCs-HUVECs were incubated at 37 °C, 5% CO_2_ in a humidified atmosphere and vascular network photographed at 8–10 h later using a camera equipped microscope (Nikon) and quantified using Image J angiogenesis analysis software. In particular, we determined and compared on pictures taken at 48 h, the Mesh index (total master segments length/number of master segments) and the percentage of retention of total tube length of HUVEC alone and in co-culture with DPSCs.

### 2.15. Statistical Analysis

All data were generated from three independent experiments and expressed as means ± standard deviation (SD). One-way analysis of variance with Dunnett’ s correction was used to assess differences among groups. Statistical significance was set at *p* ≤ 0.05

## 3. Results

### 3.1. MG Induces Glycative Stress in DPSCs

**Figure 1:**
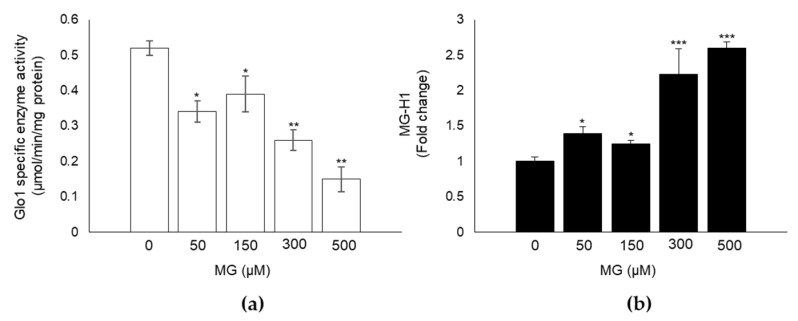
MG induces glycative stress in DPSCs. Exposure of cells to the indicated MG concentrations for 24 h induced a significant (**a**) inhibitory effect on Glo1 specific enzyme activity and (**b**) increase in MG-H1 intracellular levels. Histograms indicate the means ± SD of three different cultures, each of which was tested in triplicate. * *p* < 0.05, ** *p* < 0.01, and *** *p* < 0.001 compared with control untreated cells.

Glycative stress is a metabolic condition characterized by an abnormal high concentration of MG-derived AGEs, such as MG-H1 [1]. This condition is mostly due to a dysfunction of its main metabolizing enzyme, Glo1 [4,6,7]. Since the effect of MG, as well as the MG/Glo1 axis, on DPSC has never been studied before, we first investigated whether exposure of DPSCs to MG impaired Glo1 enzyme specific activity and led to the accumulation of MG-H1. As expected, exposure to 50, 150, 300 and 500 µM MG for 24 h induced a significant inhibitory effect on Glo1 enzyme activity levels in MG-treated compared to untreated control cells, starting from the concentration of 50 µM (Figure 1a). Moreover, immunodetection of MG-H1 in total protein extracts from both control and MG exposed DPSCs, showed that MG induced a parallel significant increase in MG-H1 intracellular levels (Figure 1b). These results suggest that MG induces glycative stress in DPSCs.

### 3.2. MG Induces Oxidative Stress in DPSCs and Impairs Cell Growth

**Figure 2:**
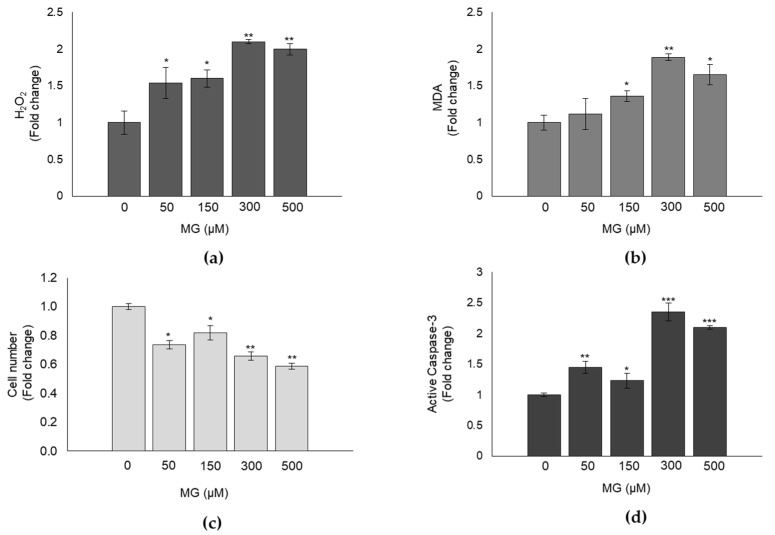
MG induces oxidative stress in DPSCs and impairs cell growth. (**a**) Hydrogen peroxide (H_2_O_2_) and (**b**) malondialdehyde (MDA) intracellular levels of untreated and MG-treated DPSCs. (**c**) MG determined a significant cytotoxic effect already at 50 μM concomitantly with (**d**) a significant increase of active caspase-3 expression. Histograms indicate the means ± SD of three different cultures, each of which was tested in triplicate. * *p* < 0.05, ** *p* < 0.01, and *** *p* < 0.001, significantly different from control untreated cells.

It is known that MG, directly or through MG-H1, is able to induce oxidative stress (OxS) and affect cell growth via cell proliferation and apoptosis control in different human cells [4,35]. Hence, we wanted to see whether a similar response was observed also in MG-treated DPSCs. As shown in Figure 2, compared to control, MG caused a significant increase in the intracellular levels of hydrogen peroxide (H_2_O_2_) (Figure 2a) and malondialdehyde (MDA) (Figure 2b), both known OxS markers, and a cytotoxic effect already at 50 μM (Figure 2c), concomitant with a significant increase of active caspase-3, the final effector of caspase-dependent apoptosis (Figure 2d). Altogether, these findings prove that MG exerts a pro-oxidant and cytotoxic effect, occurring through apoptosis induction, also on DPSCs.

### 3.3. OP Prevents MG-Dependent Dicarbonyl Stress in DPSCs

**Figure 3:**
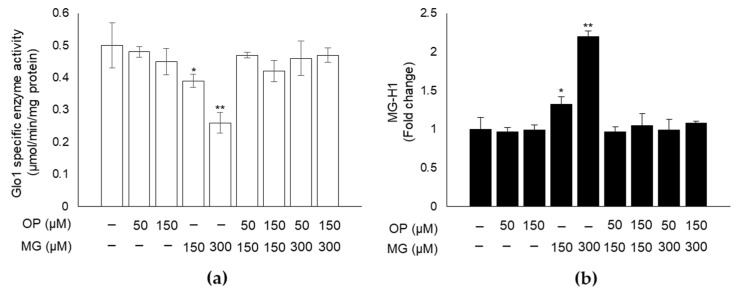
OP prevents MG-dependent dicarbonyl stress in DPSCs. (**a**) OP pretreatment for 24 h was able to significantly revert Glo1 specific activity inhibition and (**b**) MG-H1 intracellular content to the level of control. Histograms indicate the means ± SD of three different cultures, each of which was tested in triplicate. * *p* < 0.05, ** *p* < 0.01, significantly different from control untreated cells.

Emerging evidence indicates that OP is endowed with antioxidant and antiapoptotic properties [31,35]. A protective effect of OP against glycative stress has never been investigated. Hence, here we studied whether OP could induce the rescue of MG-triggered dicarbonyl stress. Indeed, we found that pretreatment of DPSCs with OP showed its ability to significantly revert Glo1 specific activity inhibition (Figure 3a) and MG-H1 intracellular content (Figure 3b) to the level of control, suggesting that OP is able to prevent MG-driven dicarbonyl stress.

### 3.4. OP Prevents MG-Dependent Oxidative Stress and Cell Growth Impairment in DPSCs

**Figure 4:**
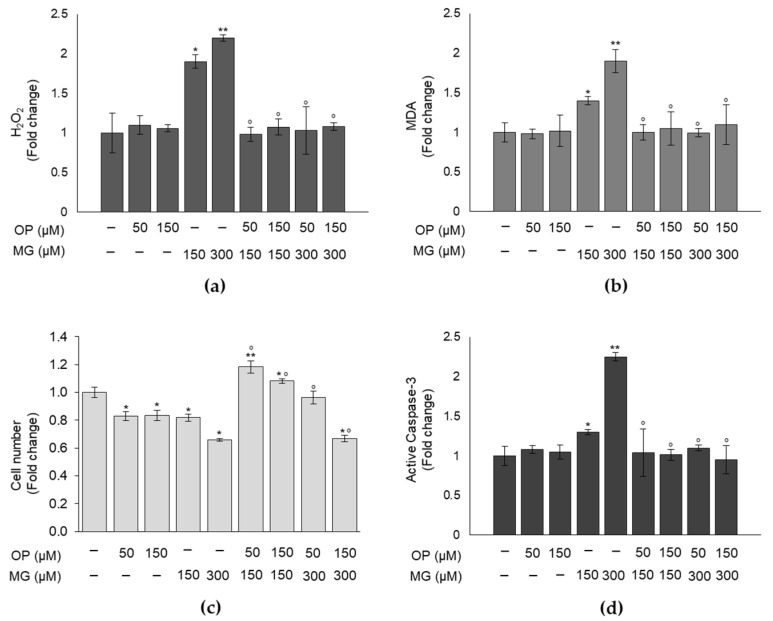
OP prevents MG-dependent oxidative stress and cell growth impairment in DPSCs. (**a**) Hydrogen peroxide (H_2_O_2_) and (**b**) malondialdehyde (MDA) intracellular levels of MG-treated DPSCs were returned by OP pretreatment to physiological levels. Similarly, OP pretreatment reverted MG-induced cytotoxic effect on (**c**) viability and the increased level of (**d**) apoptosis, evaluated by active caspase-3 measurement. Histograms indicate the means ± SD of three different cultures, each of which was tested in triplicate. * *p* < 0.05 and ** *p* < 0.01, significantly different from control untreated cells. ° *p* < 0.05 compared to MG-treated cells.

We then investigated whether OP protective function against MG-dependent glycative stress occurred also against MG-driven oxidative stress and cell growth. As expected, OP was able to return the increased levels of both H_2_O_2_ (Figure 4a) and MDA (Figure 4b), caused by MG treatment, to physiological levels. Similarly, OP pretreatment reverted the reduction of cell viability (Figure 4c) and the increase of apoptosis (Figure 4d) induced by MG. These results demonstrate that OP prevents MG-driven oxidative stress and cell growth impairment in DPSCs.

### 3.5. OP Prevents DPSCs Exposed to MG from Affecting Tube Formation of HUVECs

**Figure 5:**
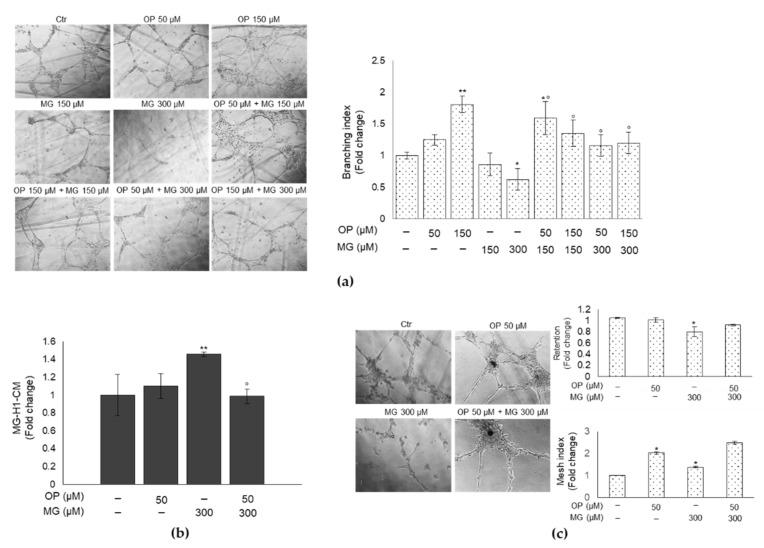
OP prevents DPSCs exposed to MG from affecting tube formation of HUVECs. (**a**) Representative images of HUVECs exposed to conditioned medium (CM) from DPSCs untreated and treated with 150 and 300 µM MG, OP 50 and 150 µM alone or combined. MG determined a reduction of the number of tubules formed (significant at 300 µM), as represented by analysis of the branching index (a measure of the number of junctions formed per vessel area). This effect was prevented by OP pretreatment both at 50 and 150 µM. This finding was positively correlated with (**b**) MG-H1 levels in CM from DPSCs treated with 300 µM MG that contained markedly higher levels of MG-H1 compared with control cells, while these levels were significantly lower in the CM of DPSCs pretreated with 50 µM OP. (**c**) Representative images of co-cultures of DPSCs and HUVECs exposed to 300 µM MG, OP 50 alone or combined. MG determined a reduction of tubules stabilization as represented by analysis of % of retention and mesh index. This effect was reverted by OP. Histograms indicate the means ± SD of three different cultures, each of which was tested in triplicate. * *p* < 0.05 and ** *p* < 0.01, significantly different from control untreated cells. ° *p* < 0.05, compared to MG-treated cells.

DPSCs are endowed of a great angiogenic and vasculogenic potential [36]. They contribute to angiogenesis during pulp regeneration by a paracrine effect and facilitate in vitro and in vivo endothelial tubulogenesis. Conversely, it has been demonstrated that MG-dependent glycation can impair angiogenesis and induce endothelial dysfunction [37]. In this light, we wanted to investigate whether conditioned media (CM) from DPSCs exposed or not to MG impaired the process of tubule formation and whether OP could prevent this response. To this aim, HUVECs were exposed to CM from MG-treated or OP-pretreated and then MG-treated DPSCs, or controls. We found that CM from DPSCs treated with 300 µM MG induced a significant reduction of the number of tubules formed, as shown by the decrease of the branching index (a measure of the number of junctions formed per vessel area), and that this effect was prevented by OP pretreatment both at 50 and 150 µM (Figure 5a). This finding was positively correlated with MG-H1 levels in CM. In fact, CM from DPSCs treated with 300 µM MG, which was able to greatly impair HUVECs tubule organization, contained markedly higher levels of MG-H1 compared with control cells, while these levels were significantly lower in the CM of DPSCs pretreated with 50 µM OP (Figure 5b). These results were also confirmed by co-culture experiments between DPSCs and HUVECs cells (Figure 5c). As recently demonstrated [27], DPSCs can support endothelial cells in forming and stabilizing vascular structures. Therefore, we evaluated the effect of DPSCs treated with 300 µM MG on HUVECs tubules formation and we observed a rescue of deleterious effect exerted by MG when DPSCs were co-treated with OP. Quantitative analysis of tube formation was performed considering the Mesh index (total master segments length/number of master segments) and the % of retention of total tube length of HUVEC in co-culture with DPSCs treated with MG, OP alone or in combination. Overall, these results suggest that DPSCs challenged with MG can release MG-H1 in the medium and, possibly, interfere through it, with the formation of tubules in HUVECs, and that OP can prevent all this, thus maintaining DPSCs angiogenic and vasculogenic potential.

### 3.6. OP Partially Protects DPSCs from MG-Induced Inflammation

**Figure 6:**
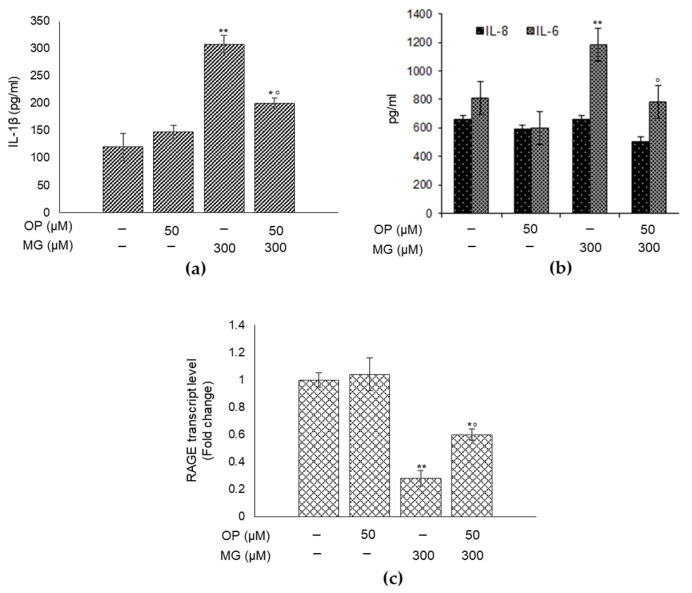
OP partially protects dental pulp stem cells DPSCs from MG-induced inflammation. The supernatants were collected, and IL-1β (**a**), IL-6 (**b**) and IL-8 (**b**) release were measured by ELISA. MG caused a significant increase in the levels of IL-1β (**a**) and IL-6 (**b**), while it dramatically reduced RAGE mRNA expression (**c**). When DPSCs were pretreated with OP, MG effects on both interleukins (**a**,**b**) and RAGE level (**c**) were reverted to that of control. Histograms indicate the means ± SD of three different cultures, each of which was tested in triplicate. * *p* < 0.05 and ** *p* < 0.01, significantly different from control untreated cells. ° *p* < 0.05, compared to MG-treated cells.

MG, mainly through MG-H1, which serves as a ligand for the receptor of AGEs (RAGE), is also an important pro-inflammatory agent [3]. Hence, we first investigated whether MG could induce inflammation in DPSCs, through the measurement of the levels of the major pro-inflammatory cytokines IL-1β, IL-6 and IL-8, and whether this biological response was paralleled by changes in RAGE mRNA expression. As shown in Figure 6, MG caused a significant increase in the levels of IL-1β (Figure 6a) and IL-6 (Figure 6b), while it dramatically reduced RAGE mRNA expression (Figure 6c). Then, we saw whether OP could prevent MG-induced increase of IL-1β and IL-6 and decrease of RAGE expression, finding that OP could revert both interleukins (Figure 6a,b) and RAGE level (Figure 6c) to that of control, even though only in part. A similar trend was observed for the IL-8, although not significant. Altogether, these results further support the pro-inflammatory role of MG also in DPSCs, where it was never investigated before, with the specific participation of IL-1β and IL-6, among those considered in this study. In addition, they show that OP is only in part involved in the control of MG-dependent pro-inflammatory role in DPSCs, keeping open the option that other mechanisms may be involved in this response. Unusually and interestingly, RAGE transcript levels were markedly decreased by MG exposure and partially rescued by OP, suggesting that MG-driven proinflammatory effect and/or the action of OP either did not occur through RAGE or, more likely, in a way that deserves further investigation.

### 3.7. MG-Induced Responses and Their Prevention by OP Are Paralleled by Changes of the Master Redox-Sensitive Transcriptional Regulator Nrf2 in DPSCs

**Figure 7:**
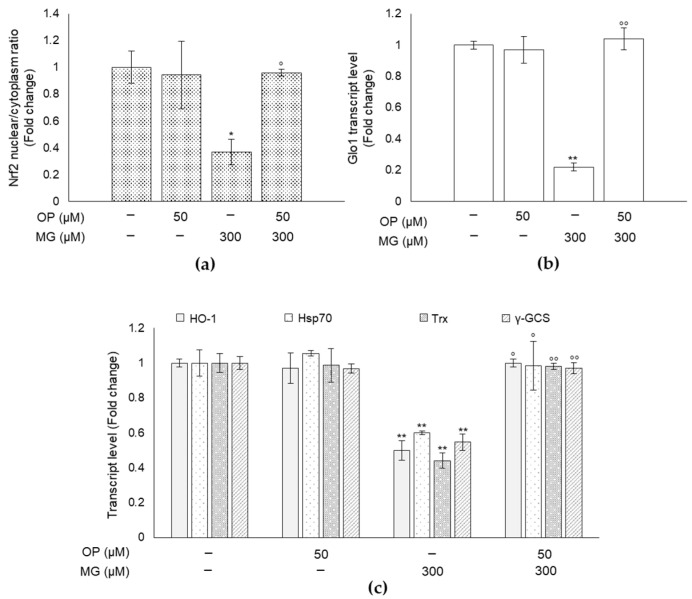
MG-induced responses and their prevention by OP are paralleled by changes of the master redox-sensitive transcriptional regulator Nrf2 in DPSCs. Nuclear and cytoplasmic extract of MG-treated DPSCs and controls were analyzed by ELISA to determine the activation of Nrf2. We observed that (**a**) the nuclear to cytoplasmic ratio of Nrf2 was significantly lower in MG-exposed DPSCs than in control cells and this decreased nuclear translocation of Nrf2 in MG-treated DPSCs was paralleled by (**b**) the downregulation of Glo1 mRNA. (**c**) Transcript levels of HO-1, Hsp70, Trx, and γ-GCS were determined after MG treatment for 24 h and in OP pretreated DPSCs. MG determined a down-regulation of HO-1, Hsp70, Trx, γ-GCS at mRNA level that was prevented in OP pretreated DPSCs Histograms indicate the means ± SD of three different cultures, each of which was tested in triplicate. * *p* < 0.05, ** *p* < 0.01 versus unexposed cells. ° *p* < 0.05, °° *p* < 0.01 compared to MG-treated cells.

To clarify the molecular mechanism underlying MG-driven responses and OP preventive action, we investigated the possible involvement of Nrf2, the master redox-sensitive transcription factor that helps cells to adapt to oxidative stress and inflammation by inducing the expression of a large number of cytoprotective genes [38], including Glo1 [39,40] and those belonging to the vitagenes network, such as members of the Heat Shock Proteins (HSP) family Heme-oxygenase-1 (HO-1, Hsp70), thioredoxin (Trx), gamma-glutamylcysteine synthetase (γ-GCS) and superoxide dismutase (SOD) [41]. To this end, nuclear and cytoplasmic extracts of MG-treated DPSCs and controls were analyzed to examine the activation of Nrf2 by ELISA assessment of its nuclear translocation. The outcomes of these experiments demonstrated that the nuclear to cytoplasmic ratio of Nrf2 was significantly lower in MG-exposed DPSCs than in control cells (Figure 7a), suggesting that Nrf2 signaling is desensitized in response to MG. Furthermore, consistent with the established transcriptional control of Glo1 by Nrf2, the decreased nuclear translocation of Nrf2 in MG-treated DPSCs was paralleled by the downregulation of Glo1 mRNA (Figure 7b), suggesting that MG exposure sustained downregulation of the Nrf2-Glo1 pathway. As expected, we observed a significant downregulation of HO-1, Hsp70, Trx, γ-GCS at mRNA level (Figure 7c) that confirmed Nrf2 signaling desensitization by MG. Among the analyzed genes, however, only SOD transcript levels were not affected by MG (data not shown). Confirming our hypothesis about the involvement of Nrf2 pathway in OP protective effect against MG-induced dicarbonyl stress, we found that OP prevented MG-induced effects by inducing the rescue of Nrf2, Glo1 and vitagenes mRNAs (Figure 7), suggesting that this secoiridoid might play an important protective role against MG-induced detrimental effects by sustaining Nrf2/Glo1 axis.

### 3.8. Pharmacological Activation of Nrf2 Confirms the Role of This Transcription Factor in MG-Induced Responses and Their Prevention by OP Through Nrf2/Glo1 Axis in DPSCs

**Figure 8:**
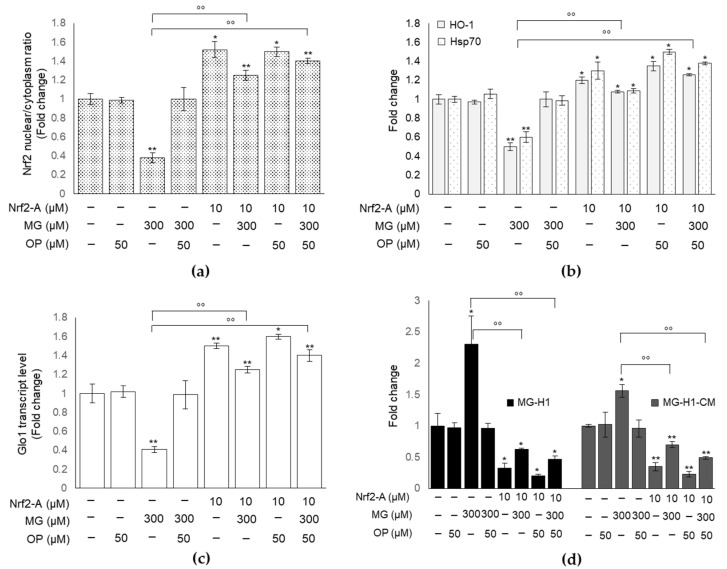
MG-induced responses and OP prevention through Nrf2/Glo1 axis in DPSCs. (**a**,**b**) Nrf2 activation resulted in (**c**) a significant rescue of Glo1 expression and reduction in (**d**) MG-H1 intracellular and released levels (MG-H1-CM). Histograms indicate the means ± SD of three different cultures, each of which was tested in triplicate. * *p* < 0.05, ** *p* < 0.01 versus unexposed cells; °° *p* < 0.01, versus MG-treated cells.

**Figure 9:**
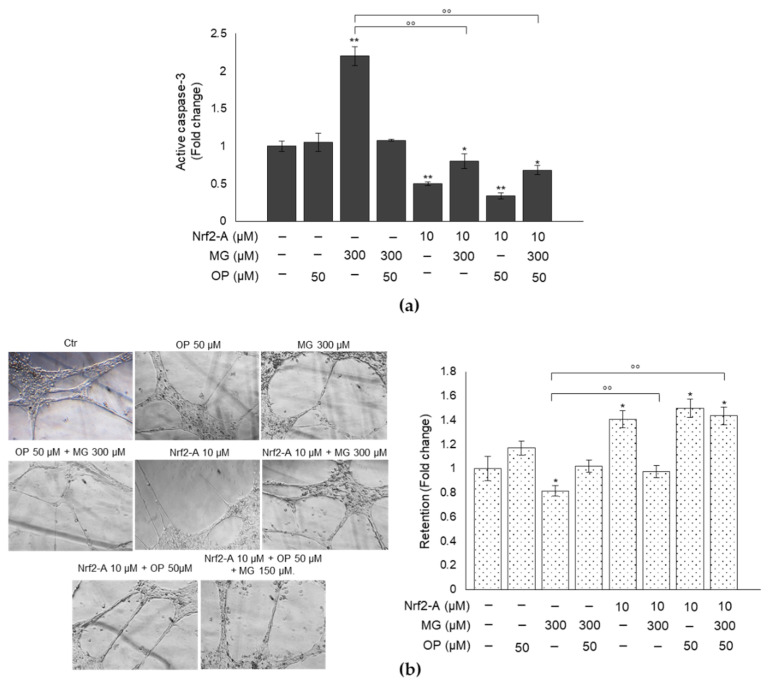
OP prevention of MG-induced responses through Nrf2/Glo1 axis in DPSCs. (**a**) Nrf2 activator (Nrf2-A) reduced MG-induced caspase-3 activation. (**b**) Representative images of co-cultures of DPSCs and HUVECs exposed to MG in the presence of OP and/or Nrf2-A show that Nrf2 activation determined a significant rescue of tubule stabilization from DPSCs, compared with MG-challenged cells. Histograms indicate the means ± SD of three different cultures, each of which was tested in triplicate. * *p* < 0.05, ** *p* < 0.01 versus unexposed cells; °° *p* < 0.05, versus MG-treated cells.

To prove that MG-induced responses as well as OP protective role against them occurred through Nrf2/Glo1 axis, we pretreated cells with a Nrf2 activator (Nrf2-A) [42], before exposing them to MG and OP, and evaluated Glo1 expression, MG-H1 intracellular or released levels, caspase-3 activation and tubule formation. We found that Nrf2 activation (Figure 8a,b) resulted in a significant rescue of Glo1 expression (Figure 8c), reduced MG-H1 intracellular accumulation and extracellular levels (Figure 8d), reduced caspase-3 activation (Figure 9a) and restored tubule formation (Figure 9b) compared with MG-challenged cells. More importantly, these effects were potentiated under OP treatment (Figure 8 and Figure 9).

Overall, these results indicate that MG-induced dicarbonyl stress and associated responses occurred through desensitization of Nrf2/Glo1 axis and that OP could prevent these detrimental effects by restoring this pathway. The use of Nrf2 activator did not affect H_2_O_2_ or MDA levels (data not shown), suggesting that OxS very likely acts upstream of the Nrf2/Glo1 axis.

### 3.9. MG-Induced Responses and Their Prevention by OP Are Paralleled by Changes of GSH in DPSCs

**Figure 10:**
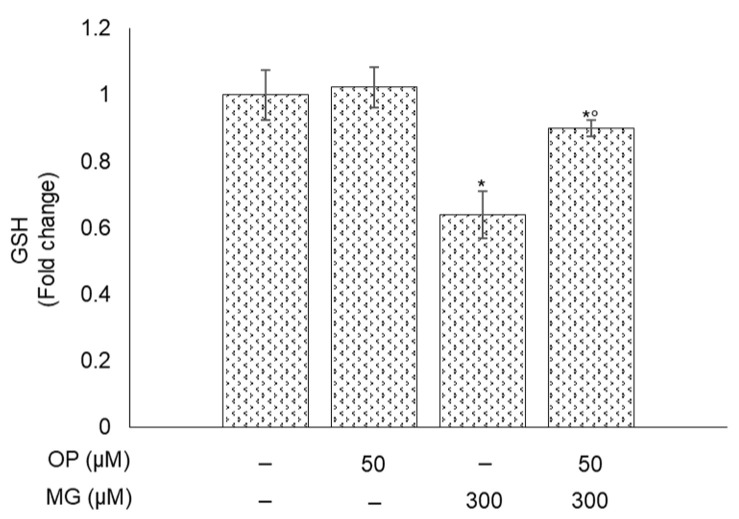
MG-induced responses and their prevention by OP are paralleled by changes of GSH in DPSCs. MG determined a reduction in GSH levels that was prevented by OP pretreatment. Histograms indicate the means ± SD of three different cultures, each of which was tested in triplicate. * *p* < 0.05, significantly different from control untreated cells. ° *p* < 0.05, compared to MG-treated cells.

GSH is an important co-factor for Glo1 activity to control MG levels [1]. Here, we found that MG treatment decreased the transcript levels of Nrf2-dependent γ-GCS, the first enzyme catalyzing the rate limiting step of the cellular GSH biosynthetic pathway, while OP prevented this down-regulation (Figure 7c). Hence, we investigated whether the observed MG-driven responses and OP preventive action might involve also GSH availability. As shown in Figure 10, GSH levels in DPSCs exposed to MG were significantly decreased compared to controls; however, this reduction was only in part prevented by OP, thus suggesting that GSH plays a marginal role in the observed results.

## 4. Discussion

In the present work, we have shown for the first time that OP prevents MG-triggered glycative stress in DPSCs by inducing the rescue of Nrf2/Glo1 axis. To reach this conclusion we first investigated whether MG could induce glycative stress in this cell model, where it had never been studied before, by evaluating the functionality of Glo1, the major MG scavenging enzyme, and the intracellular accumulation of MG-H1, the prevalent AGE originating from MG adduction of protein arginine residues [1].

In line with the results obtained in other cell models [43,44], we found that exposure to MG induced a significant inhibition of Glo1 activity compared with untreated cells, also in DPSCs. Accordingly, this was paralleled by a marked increase in MG-H1 intracellular levels, thus indicating occurrence of glycative stress in DPSCs treated with MG. Aberrant MG-H1 accumulation drives several biological responses, including oxidative stress (OxS) and apoptosis. In good agreement with previous findings [4,45], the observed glycative stress in DPSCs was paralleled by the onset of OxS, as shown by the increase in H_2_O_2_ and MDA levels, and of apoptosis, as indicated by the activation of caspase-3.

Undifferentiated mesenchymal stem cells are one of the most important components in dental pulp tissues [46], and the most important contributors to odontogenic regeneration [47,48] and repairing and regeneration of many other dental tissues [49]. Hence, MG-dependent glycative stress that, as observed here, impairs DPSC viability may have a crucial negative impact on the above-mentioned processes, with important pathological consequences. Additionally, mounting in vitro evidence, using cell models from gingival connective tissue, suggests that MG and MG-derived AGEs participate to the pathogenesis of some oral diseases, such as gingivitis and periodontitis [18,19], and some in vivo studies report the accumulation of MG in some biological fluids from gingival crevicular fluid of chronic periodontitis patients [20], where they can be conveyed by bacterial infections [20,21]. Moreover, MG is an endogenous metabolite but also a compound that can be introduced from some foodstuffs (e.g., milk, coffee, bread, fruit juices), food cooking, cigarette smoke or aerosols from nicotine delivery systems (NDS) [22,23,24]. Given that the oral cave is the gateway for these substances into our body, knowledge of the effects generated by agents favoring glycative stress is important in order to prevent oral tissues damage. Similarly, MG and MG-derived AGEs may be important contributing factors to the dental pulp pain that often appears in diabetic patients (diabetic odontalgia) [25,26], being circulating and tissue MG levels very higher in this human metabolic pathology [50]. These high levels of MG and MG-AGEs could even be responsible of the failure rate of some treatments used for curing oral cave diseases such as apical periodontitis particularly evident in patients with diabetes [51,52]. Hence, our study is also important in the area of basic pulp biology, and preventing MG-AGEs formation may be useful to relieve these clinical problems in patients with diabetes.

Notably, due to their extremely simple gathering and high proliferative potential, DPSCs have enormously broadened the horizons of regenerative medicine [27,48,53]. Hence, we would like to point out that pathophysiological conditions favoring MG accumulation, and consequently MG-derived glycative stress onset [14,15], may also have profound negative effects of paramount clinical and translational importance from this perspective.

In consideration of the great potential of DPSCs and the detrimental effects of MG-glycative stress on them, avoiding accumulation of MG and/or MG-derived AGEs is critical in both the prevention and treatment of oral diseases.

OP is a polyphenolic compound obtained from olive trees that is endowed with high antioxidant capacity [54]. It is considered one of the main active compounds of olive-tree products due to its abundance and biological activity. Indeed, it also has anti-inflammatory, anti-atherogenic, anti-tumoral, antimicrobial and neuroprotective effects, among others. Thus, OP offers numerous health benefits; so much so, that it is a potential candidate molecule for the prevention and treatment of such diseases [31,55,56]. We observed for the first time that OP is also an excellent defense against MG-dependent glycative stress in DPSCs, thus posing it as a potential candidate in dicarbonyl/glycative stress-related oral disease prevention.

Among their pleiotropic functions, DPSCs are able to facilitate in vitro and in vivo endothelial tubulogenesis [36,37,57,58]. In contrast, MG-dependent glycation impairs angiogenesis and induce endothelial dysfunction [37]. We observed that conditioned media (CM) from DPSCs exposed to MG containing high levels of secreted MG-H1, compared with untreated cells, impaired the process of tubule formation of HUVEC cells, and this effect was prevented by OP treatment. These findings, also confirmed by co-culture experiments, suggest that the intracellular MG-H1 can also be released by cells in the medium and trigger the observed response in tubules architecture in a paracrine way. When OP prevents MG-H1 accumulation in CM, tubule organization is restored. Thus, MG is not only able to reduce DPSCs viability through MG-H1 accumulation, consequent to Glo1 depleted enzyme activity, it is also able to negatively impact DPSCs’ ability to support endothelial cells in forming and stabilizing vascular structures.

MG, mainly through MG-H1, which serves as a ligand for RAGE, is also an important pro-inflammatory agent [3,59]. Consistent with the results of other studies in other cell models, we found that MG, via its AGE MG-H1, also played a pro-inflammatory role in DPSCs, particularly by inducing IL-1β and IL-6. However, while in some of these studies the AGE-induced inflammatory responses occurred via RAGE signaling, unexpectedly, this response in our study was paralleled by a dramatic decrease in RAGE mRNA expression, suggesting that MG-driven proinflammatory effect might not occur through RAGE, or occurs in a way that deserves further investigation. Additionally, AGEs can also act independently from RAGE [60].

We next showed that MG-driven responses and OP preventive action on them were mediated by Nrf2 signaling. In particular, MG induced Nrf2 signaling desensitization, also confirmed by the decreased mRNA expression of typical target genes [61], the so-called “vitagenes” (HO-1, Hsp70 and Trx), for their capacity to counteract, either individually or in concert, ROS-mediated damage [62]. More importantly, under MG treatment, the use of a specific Nrf2 activator reverted MG-driven effects on Glo1 activity, MG-H1 intracellular or released levels, caspase-3 activation and tubule formation. These effects were potentiated under OP treatment, suggesting that this secoiridoid plays a protective role against MG-induced detrimental effects by sustaining Nrf2/Glo1 axis. Hence, in agreement with other findings, we confirm that OP is a good activator of Nrf2 signaling [63,64]. Conversely, Nrf2 activator did not affect H_2_O_2_ or MDA levels. These results, together with the capacity of the antioxidant OP to revert MG-induced OxS onset, suggest that OxS very likely acts upstream of the Nrf2/Glo1/MG-H1 axis. In other words, MG induces OxS [65,66], which, in turn, desensitizes Nrf2 pathway and downstream Glo1 with the consequent accumulation/release of MG-H1, eventually leading to DPSCs apoptosis and dysfunction (impaired tubule formation). Additionally, although Nrf2 signaling represents a key cellular defense against mild to moderate OxS [67], excessive production of ROS can provoke its downregulation [68,69], as observed here. In addition, it has been reported that AGEs themselves can inhibit Nrf2 pathway, in part through ROS [68]; hence, it is reasonable to assume that intracellular MG-H1 could contribute to generate an over-amount of H_2_O_2_ and possibly other ROS, in addition to those yielded by MG itself, thus acting as a part of a positive feed forward loop within a precise regulatory circuitry.

OP is also very effective as an anti-inflammatory natural compound [70]. However, our results indicate that in DPSCs this natural compound is only partially effective in rescuing MG-induced inflammation, reinforcing the major antioxidant and antiglycation function of OP in DPSCs.

Finally, we would also like to point out the partial contribute of GSH to MG and OP-induced responses.

## 5. Conclusions

**Figure 11:**
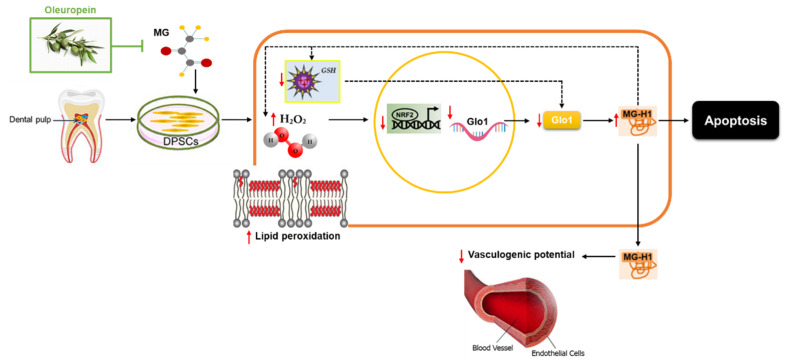
Proposed mechanism for MG and OP effect on DPSCs. MG would induce oxidative stress (increase in hydrogen peroxide, H_2_O_2_, and malondialdehyde, MDA) that, in turn, desensitizes Nrf2 pathway and down-stream Glo1with the consequent accumulation/release of MG-H1 eventually leading to DPSCs apoptosis and dysfunction (impaired tubule formation). OP, by preventing this mechanism, plays a crucial protective role from MG damage in DPSCs. Intracellular MG-H1 could contribute to generating an excess of H_2_O_2_ and possibly other ROS, in addition to those yielded by MG itself, thus acting as a part of a positive feed forward loop within a precise regulatory circuitry (dotted line). MG might deplete GSH which could contribute to impair Glo1 activity (dotted line).

The present results provide strong evidence that OP possesses a protective activity against MG-induced cytotoxicity/dysfunction in human DPSCs by preventing MG-induced ROS-mediated glycative stress through a mechanism involving Nrf2/Glo1 axis (Figure 11).

We suggest that OP holds great promise for the development of preventive strategies for AGEs-associated oral diseases. Moreover, being an easily accessible natural antioxidant, we put forward the hypothesis of its use as an ingredient for functional food and pharmaceutical agents.

## Data Availability

The data presented in this study are available on request from the corresponding author.
